# Vasculogenic Mimicry: A Promising Prognosticator in Head and Neck Squamous Cell Carcinoma and Esophageal Cancer? A Systematic Review and Meta-Analysis

**DOI:** 10.3390/cells9020507

**Published:** 2020-02-24

**Authors:** Roosa Hujanen, Rabeia Almahmoudi, Sini Karinen, Bright I. Nwaru, Tuula Salo, Abdelhakim Salem

**Affiliations:** 1 Department of Oral and Maxillofacial Diseases, Clinicum, University of Helsinki, 00014 Helsinki, Finland; 2 Krefting Research Centre, Institute of Medicine, University of Gothenburg, 40530 Gothenburg, Sweden; 3 Wallenberg Centre for Molecular and Translational Medicine, Institute of Medicine, University of Gothenburg, 40530 Gothenburg, Sweden; 4 Translational Immunology Research Program (TRIMM), Research Program Unit (RPU), University of Helsinki, 00014 Helsinki, Finland; 5 Cancer and Translational Medicine Research Unit, University of Oulu, 90014 Oulu, Finland; 6 Medical Research Centre, Oulu University Hospital, 90220 Oulu, Finland; 7 Helsinki University Hospital (HUS), 00029 Helsinki, Finland

**Keywords:** meta-analysis, prognosis, vasculogenic mimicry, cancer cell-lined vessels, head and neck squamous cell carcinoma, esophageal squamous cell carcinoma

## Abstract

Vasculogenic mimicry (VM) is an intratumoral microcirculation pattern formed by aggressive cancer cells, which mediates tumor growth. In this study, we compiled the evidence from studies evaluating whether positive VM status can serve as a prognostic factor to patients with squamous cell carcinoma of the head and neck (HNSCC) or esophagus (ESCC). Comprehensive systematic searches were conducted using Cochrane Library, Ovid Medline, PubMed, and Scopus databases. We appraised the quality of studies and the potential for bias, and performed random-effect meta-analysis to assess the prognostic impact of VM on the overall survival (OS). Seven studies with 990 patients were eligible, where VM was detected in 34.24% of patients. Positive-VM was strongly associated with poor OS (hazard ratio = 0.50; 95% confidence interval: 0.38–0.64), which remained consistent following the subgroup analysis of the studies. Furthermore, VM was associated with more metastasis to local lymph nodes and more advanced stages of HNSCC and ESCC. In conclusion, this study provides clear evidence showing that VM could serve as a promising prognosticator for patients with either HNSCC or ESCC. Further studies are warranted to assess how VM can be implemented as a reliable staging element in clinical practice and whether it could provide a new target for therapeutic intervention.

## 1. Introduction

Head and neck squamous cell carcinoma (HNSCC) remains one of the most common and deadly cancers worldwide [1]. HNSCC encompasses a group of aggressive tumors that occur throughout the oral cavity, hypopharynx, oropharynx, nasopharynx, or larynx [2,3]. They primarily develop through chemically induced (i.e., tobacco and alcohol abuse) or virally induced (i.e., human papillomavirus) carcinogenesis [2,4]. The incidence and mortality of HNSCC are rapidly growing in different geographic regions, including many European and Nordic countries [5]. Some variants of HNSCC, such as basaloid SCC, are characterized by a biphasic pattern of growth and have an aggressive clinical behavior [6]. Esophageal cancer ranks seventh in terms of incidence and sixth in mortality [1]. Several studies have shown that HNSCC is often accompanied by esophageal squamous cell carcinoma (ESCC) [7]. HNSCC and ESCC share broad similarities, such as cellular origin and tumorigenesis, in addition to their early dissemination and dismal prognosis [2,8].

Cancer metastasis is the main leading cause of cancer-associated morbidity and mortality, which accounts for about 90% of cancer-specific deaths [9]. In spite of the multimodality approach in the management of HNSCC patients, the overall survival (OS) rate is low, particularly when presented with lymph node metastasis (LNM) and/or recurrence [3,10]. Indeed, a better understanding of cancer biology is essential to develop more effective treatments that will target, for instance, the mechanisms underlying tumor growth and metastasis.

Vasculogenic mimicry (VM) was first introduced in 1999 to indicate a remarkable feature of aggressive melanoma cells to generate vessel-like, blood-containing channels which facilitate tumor perfusion regardless of surrounding endothelial blood vessels [11,12]. Clinically, VM is commonly assessed in patient samples by immunohistochemical (IHC) analysis of periodic acid–Schiff (PAS) positive, and CD31 or CD34 negative vessel-like structures [13]. PAS stains basement membranes while CD31 and CD34 are regarded as endothelial cell markers. The importance of VM stems from the ability of certain rapidly-growing tumors to link fulfilment of the nutritional needs with metastatic progression [14,15]. VM significantly associates with worse prognosis in cancer patients, and hence it represents an attractive target for novel anticancer drug discovery [14]. Moreover, VM appears to drive distant metastases of breast cancer cells and simultaneously promotes the passage of red blood cells and nutrients into the tumoral tissue [15]. However, some studies have also shown that VM is not significantly associated with cancer prognosis, albeit such patients exhibited shorter OS compared with VM-free patients [16,17]. Based on the growing interest in VM as a novel therapeutic target in cancer, clarifying its impact on prognosis will enhance its utility as a biomarker. Therefore, to address this objective, we conducted a systematic review to identify, critically appraise, and synthesize the evidence from studies that have so far investigated the association between VM and the prognosis of patients with HNSCC or ESCC. We specifically evaluated whether immunodetection of VM serves as a prognostic factor of the survival of these patients.

## 2. Materials and Methods

### 2.1. Protocol and Registration

Prior to undertaking the systematic review, we developed a protocol for the work, which was registered in the international prospective register of systematic reviews PROSPERO (https://www.crd.york.ac.uk/prospero/) with the following identification number: CRD42019139244. The protocol was based on PRISMA (Preferred Reporting Items for Systematic Reviews and Meta-Analyses) guidelines [18,19].

### 2.2. Eligibility Criteria

We included original research articles that investigated the relationship between VM and the survival outcomes of patients with HNSCC or ESCC. We excluded review articles, case reports, case series, and reports lacking survival data. The detailed inclusion and exclusion criteria are listed in Table 1.

### 2.3. Search Strategy

We conducted a comprehensive search in four databases, including Cochrane Library, Ovid Medline, PubMed, and Scopus up to June 17th, 2019, without language restriction. The following MeSH terms and keywords related to VM were applied: (“vasculogenic mimicry” OR “tumor cell-lined vessels”) AND (“head and neck neoplasms” OR “head and neck cancer” OR “head and neck squamous cell cancer” OR “esophageal cancer” OR “esophageal squamous cell cancer” OR “oral cancer” OR “mouth neoplasms” OR “laryngeal neoplasms” OR “gingival neoplasms” OR “oral leukoplakia” OR “lip neoplasms” OR “palatal neoplasms” OR “tongue neoplasms” OR “pharyngeal neoplasms” OR “squamous”). Moreover, we used exploded MeSH terms for each cancer type in Ovid Medline. The retrieved studies were imported to RefWorks and duplicates were removed. Screening of studies by title and abstract was next undertaken. Finally, full-text screening and evaluation was performed. The literature screening was undertaken independently by two reviewers (RH and RA) and a third reviewer (AS) arbitrated if there were discrepancies.

### 2.4. Data Extraction and Study Items

We developed a data extraction form, which was used to extract relevant information from each study. Data extraction was independently performed by two authors (RH and RA), and any discrepancies were resolved by consensus-based discussion with a third reviewer (AS). From each study, we extracted the following key information: first author’s name, year of publication, country, tumor information (type, size, and location), type of the samples, total number of patients, number of positive-VM samples, main findings, methods used to detect VM, antibody information, criteria used to define VM, outcome measures, and estimates of prognosis (such as hazard ratio (HR) with their respective 95% confidence interval (95% CI) and p values).

### 2.5. Assessment of Reporting Quality and Risk of Bias

The quality of reporting in included studies was assessed according to the reporting recommendations for tumor marker prognostic studies (REMARK) guidelines [20]. From the REMARK checklist, we adapted the following six essential items as they were deemed pertinent for our study: (1) patient samples; (2) clinical data; (3) immunostaining; (4) prognostic data; (5) statistical analysis; and (6) classical prognostic factors. The applied REMARK parameters are listed in Table S1. The Meta-Analysis of Statistics Assessment and Review Instrument tool (MAStARI) was utilized to evaluate the risk of bias in the included studies [21]. Ten questions were independently applied in each study by two reviewers (RH and RA). For each question, the answers were expressed as yes, no, unclear, or not applicable as illustrated in Table S2. Accordingly, the risk of bias was categorized as high (i.e., the study is carrying high risk of bias) when answers contain ≤49% of “yes” score, moderate (i.e., the study is carrying moderate risk of bias) when answers contain 50–69% of “yes” score, or low (i.e., the study is carrying low risk of bias) when answers contain ≥70% of “yes” score. The “not applicable” questions were not included when calculating the percentage of “yes” scores. Any discrepancies were resolved by discussion between three authors (RH, RA, and AS). Such discrepancies resulted from overlooking of data or due to misunderstanding of information, and thus they were easily resolved without too much discussion.

### 2.6. Data Synthesis and Statistical Analysis

We produced a descriptive table, as shown in Table 2, to summarize the characteristics as well as the quality of the included studies based on the REMARK framework [20]. We employed both narrative and quantitative synthesis to summarize the evidence from the studies. For the quantitative synthesis, we performed random-effects meta-analysis through the DerSimonian–Laird estimate of the variance of the effect sizes to pool estimates from the studies that we judged to be reasonably homogenous with regards to their consistency in methodology and definitions. The weight assigned to the studies in meta-analyses were based on the inverse variance method in which the weight given to each study contributing to the meta-analysis is the variance of the effect estimate (that is, weight = 1 divided by the square of the standard error of the effect estimate). Based on the inverse variance weighting mechanism, larger studies (usually with smaller standard errors) receive more weight than smaller studies (which have larger standard errors). Out of the seven studies included in the review, meta-analysis was possible only for four studies [22,23,24,25]. We excluded the studies by Liu et al. and Xu et al. from the meta-analyses because they did not provide effect estimates for the influence of VM on survival [26,27]. We excluded the study by Lin et al. from the meta-analyses because it estimated only disease-free survival (DFS) but not OS [28]. Secondly, it was also based on the same cohort as the study by Wang et al. [22], therefore, including it would mean the inclusion of two studies from the same participants.

To perform the meta-analyses, some recalculations were performed when necessary. For instance, some studies looked at the impact of VM positivity (using VM negativity as the reference) on survival, while some studies looked at the impact of VM negativity (using VM positivity as the reference). Therefore, in order to have a consistent independent variable and reference group for VM, we changed the reference groups for all studies that have looked at VM negativity (using VM positivity as the reference) to VM positivity (using VM negativity as the reference). We did this by dividing the value one by the HRs and the accompanying 95% CI from the relevant studies. In this way, we ensured that all studies included in the meta-analysis have the same reference group and are estimating the same effect, thus comparability of effects between studies is ensured. We performed two sets of meta-analyses: one in which the four studies were included in the same analysis; and one in which we divided the studies into two subgroups (HNSCC and ESCC). We quantified any potential heterogeneity between studies in the pooled estimates using the I^2^ statistic, which is a measure (range 0%–100%) that quantifies the percentage of variance in the pooled estimates that is attributable to differences in estimates between the meta-analyzed studies. The between-study variance was estimated using a Tau-squared (T^2^) statistic derived from the DerSimonian–Laird approach. All tests were 2-sided, and *p* < 0.05 was considered statistically significant. Analyses were performed using Stata release 14 (StataCorp. 2015. Stata Statistical Software: Release 14. StataCorp LP, College Station, TX, USA).

## 3. Results

### 3.1. Study Selection

**Figure 1:**
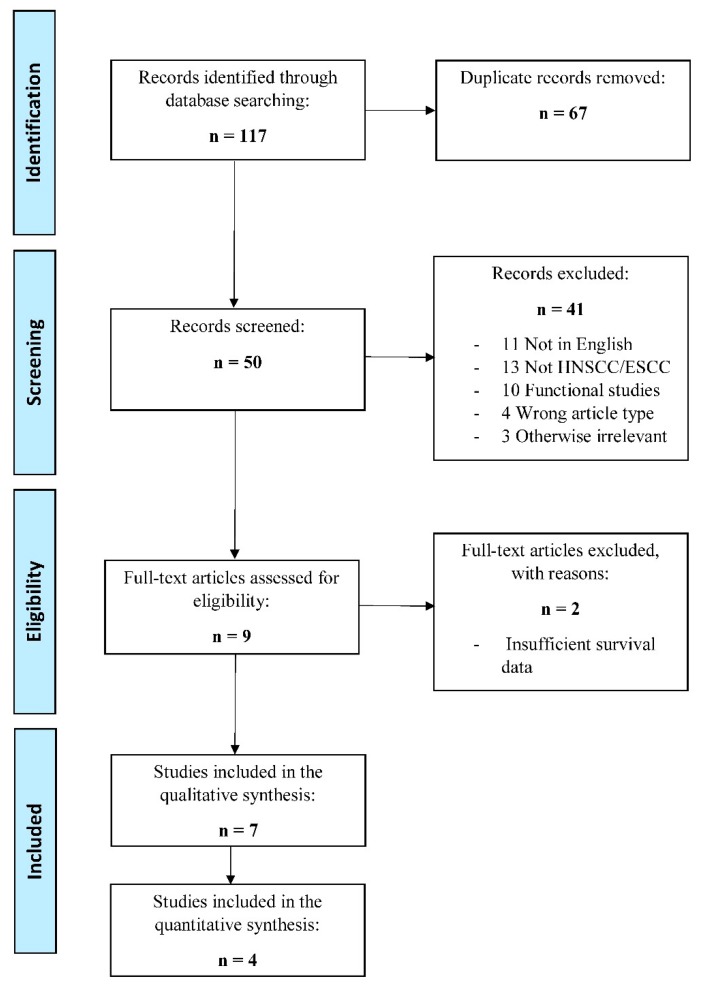
Flowchart diagram of literature search and selection. Irrelevant article types and other exclusion criteria are listed in Table 1.

In total, our database searches yielded 117 records, of which 67 were duplicate records, leaving 50 articles that further screened for their eligibility. After screening by titles and abstracts, 41 articles were excluded for not meeting the inclusion criteria. The full-texts of the remaining nine articles were subsequently screened, of which seven records were included in this systematic review, as shown in Figure 1.

### 3.2. Study Characteristics

The included articles were published between 2008 and 2018 and presented data from patients with SCC in the oral cavity proper, larynx, nasopharynx, and esophagus. The immunostaining in these studies was performed on formalin-fixed paraffin-embedded samples. The sample sizes varied from 40 to 203, with a total of 990 patients from all studies. Two studies investigated VM in ESCC patients (*n* = 277), while the other five studies included patients with HNSCC (*n* = 713). Two studies were based on the same participants and were conducted on laryngeal squamous cell carcinoma (LSCC) samples from the same recruited cohort (from January 1990 to January 2003) and in the same institution [22,28]. Characteristics of the included studies are summarized in Table 2.

### 3.3. Quality and Bias Assessment

Two studies fulfilled all of the six REMARK-adapted checklist items [23,24]. Five studies lacked one or more items as illustrated in Table 2. Based on the MAStARI assessment [21], five studies were classified as having a low risk of bias [22,23,24,25,28]. However, the study by Liu et al. was evaluated as carrying a moderate risk of bias as only 50% of the questions were addressed as “yes” [26]. One study was classified as bearing high risk of bias, mainly because they lacked critical information regarding questions 1 through 4, and thus had only a 25% “yes” score [27]. Further details can be found in Table S2.

### 3.4. Identification of VM in HNSCC/ESCC Patients

In all the studies, VM was identified by the IHC double staining method. In the first published report, authors defined VM as positive pancytokeratin, to highlight tumor cells, and negative CD34 lining [26]. In the rest of studies, VM was identified as positive-PAS and negative CD31 or CD34 lumens. In addition, three studies highlighted additional criteria using hematoxylin-eosin staining, including absence of hemorrhage, necrosis, or perivascular inflammatory cell infiltrate [22,25,28]. The presence of red blood cells in the VM-lumen was indicated by two studies [23,26]. Morphologically, the VM channels established different forms, ranging from straight, curved, or branched patterns. Importantly, such positive vessel-like structures were detected in all the studies, with a total of 339 positive-VM patients (34.24%). The VM-identification methods and antibodies are summarized in Table 3.

### 3.5. Association between VM and Clinicopathological Factors

Liu et al. revealed that VM was significantly correlated with more LNM in oral squamous cell carcinoma (OSCC) patients (*p* = 0.006) [26]. In a larger OSCC cohort, Wu et al. revealed a significantly positive correlation between VM and tumor size, grade, LNM, and TNM (tumor, node, and metastasis) stages (r = 0.447; *p* < 0.001) [25]. In the retrospective study undertaken by Wang et al., VM was significantly higher in the advanced stages (III and IV) than in the primary ones (I and II) (27.97% vs. 12.94%; 0.010). In addition, VM was more frequently observed in LSCC patients with local LNM (*p* = 0.003) as well as in the advanced histopathological grades (*p* < 0.0001) [22]. This was in agreement with a study by Lin et al., which concluded a positive correlation between VM and more advanced pTNM stages (*p* = 0.024), more LNM (*p* = 0.003), and worse histopathological grade (*p* < 0.0001) [28]. Two studies examined the correlation between VM and clinicopathological features in ESCC cohorts. Chai et al. reported significant associations between VM and positive LNM (*p* < 0.001), presence of serosa infiltration (*p* < 0.001), and more progressive pTNM stages (*p* < 0.001) [23]. However, Zhang et al. found that VM was correlated with the TNM stages (*p* = 0.003) but not with the other clinicopathological elements of ESCC patients [24].

### 3.6. Survival Endpoints

Several survival endpoints were reported in the eligible studies, as shown in Table 4. Four studies reported data regarding the association between VM and OS [22,23,24,25]. Two studies defined OS as the time from the date of first biopsy to the date of cancer-related deaths [23,24]. Chai et al. reported the OS of (VM^+^/VM^−^) in ESCC patients as 28.038/66.452 (months), while Wu et al. found the ratio in OSCC patients was 41.1(±16)/58.9(±14.5) [20,22]. DFS was reported as the primary endpoint in one study [26], and with OS in two studies [22,24]. In addition to DFS, Lin et al. also adopted metastasis-free survival and local-recurrence free survival as endpoints in their study [28].

### 3.7. Prognostic Value of VM in HNSCC/ESCC Patients

Individually, each study reported that VM positivity (compared to VM negativity) was associated with poorer survival, i.e., positive-VM individuals were more likely to die earlier than those with negative-VM status. When the studies were pooled together in a meta-analysis, the HR for OS was 0.50 (95% CI 0.38–0.64), as shown in Table 5. The I^2^ value for the heterogeneity between the studies was 0% and the *p*-value associated with this was 0.575. In the subgroup analyses, in which studies based on HNSCC and ESCC were analyzed separately, the worse OS among individuals with positive VM compared those with negative VM remained consistent, although OS was lower in the ESCC subgroup (HR 0.40; 95% CI 0.26–0.63) than in the HNSCC subgroup (HR 0.55; 95% CI 0.40–0.74), as shown in Table 5.

## 4. Discussion

This systematic review and meta-analysis summarized the results of seven clinical studies involving SCC of the head, neck, and esophagus, and represented a total of 990 patients, of which 339 (34.24%) had positive-VM status. All the studies showed that positive-VM immunoreactivity is associated with a decreased probability of overall survival, so that patients with positive-VM were more likely to die compared with negative-VM patients. This was further confirmed when we separately analyzed HNSCC and ESCC studies, as such a shorter survival rate was still evident with positive-VM patients. Furthermore, a positive VM status in these studies was commonly associated with worse prognostic clinicopathological factors, such as LNM and TNM stage.

**Figure 2:**
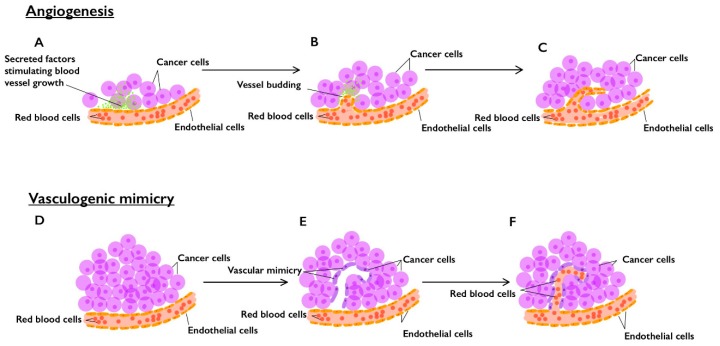
Angiogenesis versus vasculogenic mimicry (VM) in the tumorigenesis of solid tumors. In angiogenesis: (**A**) tumor cells secrete potent angiogenic factors; (**B**) these factors enhance the budding and growth of pre-existing blood vessels; (**C**) new endothelial cell-lined blood vessels are formed and enrich the tumor microenvironment. In VM: (**D**) aggressive starved tumor cells can utilize an alternative non-angiogenic vascularization method; (**E**) tumor cells start to generate new patterned vessel-like anastomoses; (**F**) these new tumor cell-lined channels invade host vessels and increase nutrient retrieval to nourish tumor tissue.

Cancer growth and distant dissemination are associated with worse prognosis and both rely on adequate blood supply [29]. Indeed, angiogenesis, which is regulated by a number of diffusible angiogenic factors, plays a vital role in promoting tumor nourishment and, subsequently, tumor development and metastasis [30,31]. VM is another non-angiogenic pattern of tumor vascularization, which refers to the ability of tumor cells to create their own vessel-like channels and to function as endothelial-like cells (ELC) [30,32]. In fact, this highlights the multidirectional extent of phenotypic plasticity in aggressive tumor cells, which is harnessed to ensure sufficient, non-lymphogenic, blood perfusion and subsequent tumor growth and metastasis [30]. We illustrate the difference between angiogenesis and VM in Figure 2.

The most important strengths of our study include the relatively low level of inter-study heterogeneity regarding VM definition, immunodetection assays, clinicopathological features, and the key findings. We had a comprehensive search of eligible studies with the search proposal and outline of the review processes developed in a protocol, which was registered in PROSPERO prior to performing the systematic review. However, we also acknowledge some limitations, such as the limited number of studies which might reflect the novelty of the VM concept. We were also compelled to exclude certain studies from the meta-analysis because they lacked the adjusted analysis or due to sample overlap [22,26,28]. Five studies did not provide all of the recommended information related to the staining protocol, which highlights the need to implement REMARK guidelines in research practice, as shown in Table 2. In addition, all the included reports were from China; hence our findings should be interpreted with caution, as they may not be widely applicable. Nevertheless, VM has been shown as a strong prognostic factor in different population segments [32]. Although the included studies provided some criteria for the immunodetection of VM, this method can be complicated by the presence of empty basement membrane remnants of regressed vessels, which may look confusingly similar to VM. Therefore, investigators are encouraged to search for new protocols to better characterize VM structures.

Indeed, our findings are in agreement with previous meta-analysis reports that have shown the association between VM positivity with decreased probability of patients’ survival in different cancers. In two recent meta-analysis studies, VM was associated with a more aggressive tumor phenotype and poor prognosis in patients with breast and gastric cancers [33,34]. In addition, Zhang et al. reported that VM status can serve as a promising prognostic biomarker in the prognosis of malignant melanoma patients [35]. In the same direction, another recent meta-analysis showed that positive VM was a reliable indicator of poor prognosis in digestive cancer patients [36]. In a larger-scale meta-analysis study that included 15 types of cancers and 3062 patients, VM-positive cancer patients show a poor 5-year OS compared with VM-negative cases [16]. Importantly, Yang et al. concluded that VM was associated with statistically significantly poorer OS in head and neck cancer patients [32]. Moreover, VM formation was regarded as an unfavorable prognostic indicator in hepatocellular carcinoma [37], osteosarcoma [38], non-small cell lung cancer [39], colorectal cancer [40], and in cutaneous melanoma [41]. Furthermore, Wang et al. reported a strong positive correlation between VM and distant metastases in salivary adenoid cystic carcinoma [42].

The mechanisms underlying such a strong association between VM status and patients’ survival are not clearly understood. However, several potential explanations were suggested, for instance, that aggressive tumors harness their own VM channels to obtain a functional perfusion pathway, independently of angiogenic vasculature, and thus enhances their progression and metastasis [15,43]. Hypoxia has also been suggested as a crucial factor in VM development [44]. In this review, two studies reported a close association between the presence of “cancer cell-lined vessels”, or VM, and hypoxia-inducible factor-1 alpha (HIF-1α) in OSCC and ESCC [23,26]. Interestingly, hypoxia was recently shown to induce epithelial–mesenchymal transition, which contributes to cancer progression via transition of epithelial cells to potent mesenchymal migratory cells, and VM formation [42,45]. Hypoxia was also reported to promote VM through other signaling pathways, such as the extravascular VE-cadherin and its role in the acquisition of the VM phenotype [46]. In addition, the multi-phenotypic reciprocity, observed in VM-forming cancer cells, has the capacity to facilitate tumor progression and metastasis [47,48]. Because of such a phenotypic switch, the VM-forming ELC are different from normal endothelial cells, rendering the VM channels inherently resistant to conventional anti-angiogenic therapy [32,49]. More interestingly, antiangiogenic treatment with Bevacizumab induced VM formation and metastasis in an ovarian cancer model [50]. In fact, antiangiogenic drugs lead to a hypoxic tumor microenvironment which enhances invasiveness and VM formation [49].

The term “mosaic vessels” was developed by Chang et al. when they noticed that some of the intratumoral VM lumens were lined by both endothelial cells and tumor cells [51]. Although such mosaic lumens were observed in less than 5% of the vascular surface area of colon cancer tissue, they were suggested as important contributors to metastasis and drug delivery [51,52,53]. It is worthy to note that none of the included studies have recognized such potential co-localization when identifying the VM structures. However, it remains unclear whether this pattern was not detected at all or was just excluded from the VM definition when designing the study.

## 5. Conclusions

This systematic review and meta-analysis provide clear evidence showing that VM could represent a promising prognosticator for patients with either HNSCC or ESCC. Moreover, VM was strongly associated with cancer differentiation, LNM, and TNM stage, which highlight its putative role in tumor growth and metastasis. Further mechanistic studies are warranted to uncover the biological processes through which VM influences survival, as well as an emerging potential therapeutic target in solid tumors. Prognostic studies on different types of populations are also required to assess how to implement VM as a reliable staging element in clinical practice.

## Figures and Tables

**Table 1:** Inclusion and exclusion criteria applied in this review.

Inclusion Criteria	Exclusion Criteria
Original research articles	• The retrieved records were: ○ case reports ○ reviews ○ letters
Human histological tissue samples	Articles in which results were based on animal models or tests
Patients diagnosed with HNSCC/ESCC	Articles in language other than English
Studies reported the association between VM expression and the survival outcomes	Insufficient information of the correlation between clinical features and/or survival outcomes

HNSCC, head and neck squamous cell carcinoma; ESCC, esophageal squamous cell carcinoma; VM, vasculogenic mimicry.

**Table 2:** Characteristics of the included studies.

Study	Country	Tumor Type	Tumor Stage/Size	No. of Cases	No. of VM^+^ Cases	Compliance to REMARK ^†^
[26]	Taiwan	OSCC	T1–T4 I–IV	112	41 (36.60%)	Lacked items No. 1–3, 5, 6
[22]	China	LSCC	T1–T4 ≤3, >3 cm	203	44 (21.67%)	Lacked item No. 3
[28]	China	LSCC	T1–T4 I–IV	168	37 (22.02%)	Lacked item No. 3
[23]	China	ESCC	I–IV	160	78 (48.75%)	Fulfilled all items
[24]	China	ESCC	I–III	117	56 (47.86%)	Fulfilled all items
[25]	China	OSCC	I–IV ≤2, ≤4, >4 cm	190	60 (31.57%)	Lacked item No. 3
[27]	China	NPC	I–IV	40	23 (57.50%)	Lacked items No. 3, 5

OSCC, oral squamous cell carcinoma; LSCC, laryngeal squamous cell carcinoma; ESCC, esophageal squamous cell carcinoma; NPC, nasopharyngeal carcinoma; VM^+^, positive vasculogenic mimicry. ^†^ The reporting quality of the eligible studies was assessed according to the REMARK guidelines [20].

**Table 3:** Summary of vasculogenic mimicry identification methods.

Study	Method	Tissue	Reagent Information	VM Definition	Additional Criteria
[26]	IHC, EnVision Doublestain	FFPE	CK: (Mo, MC), Dako CD34: (Mo, MC), Dako	CK^+^/CD34^−^ lumens	RBCs in lumens
[22]	HE, IHC double staining	FFPE	CD31: (Mo), Zhongshan Biotechnology PAS: Department of Pathology, Tianjin Hospital	CD31^−^/PAS^+^ loops around cancer cells, with/without RBCs	tumor cell-lined; no hemorrhage, necrosis, perivascular inflammatory cell infiltrate
[28]	HE, IHC double staining	FFPE	CD31: (Mo), Zhongshan Biotechnology PAS: Department of Pathology, Tianjin Hospital	CD31^−^/PAS^+^ loops around cancer cells, with/without RBCs	HE: no signs of hemorrhage, necrosis, or perivascular cell infiltrate
[23]	IHC double staining	FFPE	CD34: (Mo, MC), Abcam PAS: ND	CD34^−^/PAS^+^ vessel-like structures surrounded by tumor cells in different forms (straight, curved or branched patterns)	RBCs in channels; few necrotic/inflammatory cells near the channels
[24]	HE, IHC double staining	FFPE	CD34: (Rb, MC), Abcam PAS: ND	CD34^−^/PAS^+^ lumens composed of tumor cells	-
[25]	IHC double staining	FFPE	CD34: (Mo, MC), Abcam PAS: ND	CD34^−^/PAS^+^ small vessel-like structures	No necrosis or hemorrhage near VM
[27]	IHC double staining	FFPE	CD34: (Rb, MC), Abcam PAS: Sigma-Aldrich	CD34^−^/PAS^+^ channels with a lining of tumor cells on the external wall	No ECs on the inner wall

CK, pancytokeratin; ECs, endothelial cells; RBCs, red blood cells; Mo, mouse antihuman; Rb, rabbit antihuman; MC, monoclonal antibody; VM, vasculogenic mimicry; HE, hematoxylin-eosin staining; PAS, periodic acid–Schiff; ND, not disclosed; FFPE, formalin-fixed paraffin-embedded.

**Table 4:** Summary of the reported prognostic data and interpretation of the main findings.

Study	End-Point	Adjusted Analysis	Adjusted Factors	Results Interpretation
[26]	DFS	-	-	VM correlates significantly with poor survival
[22]	OS	HR = 2.117, *p* = 0.003	VM, recurrence, TNM stage, radiotherapy	VM is related to pTNM stage, LNM. VM adversely predicted OS and DFS
		95% CI = 1.286–3.425		
	DFS	-		
[28]	DFS	HR = 2.57, *p* = 0.003	VM, recurrence, radiation	VM was an adverse prognosticator for DFS and MFS by univariate survival analyses. VM is independent prognostic factor for only DFS
		95% CI = 1.388–4.757		
	MFS	-		
	LRFS	-		
[23]	OS	HR = 0.458, *p* = 0.04 95% CI = 0.217–0.9681	Gender, age, site, gross morphology, size, DIF, LNM, serosa infiltration, pTNM, VM, HIF-1a, E-cad	VM was significantly correlated with LNM, infiltration, pTNM staging, and 5-year OS of ESCC patients.VM is independent risk factors of patients with ESCC
[24]	OS	HR = 0.369, *p* = 0.001	pTNM, DIF, TIN expression, VM	VM indicates poor OS and DFS. VM is significant independent prognostic predictors in ESCC
		95% CI = 0.207–0.658)		
	DFS	-		
[25]	OS	HR = 1.674, *p* = 0.010 95% CI = 1.131–2.476	LGR5, VM, TNM, LNM	VM was positively related to tumor size, grades, LNM, TNM stages, and inversely with patients OS
[27]	PFS	-	-	VM formation was associated with a poor prognosis in NPC patients

DFS, disease-free survival; DIF, differentiation; ESCC, esophageal squamous cell carcinoma; LGR5, leucine-rich repeat-containing G-protein coupled receptor 5; LNM, lymph node metastasis; LRFS, local recurrence free survival; MFS, metastasis-free survival; OS, overall survival; PFS, progression-free survival; TIN, tumor-infiltrating neutrophil; VM, vasculogenic mimicry.

**Table 5:** Meta-analysis of the association between VM and OS.

Study	No. of Cases	Age Range (Years)	Age Median (Years)	Hazard Ratio (95% CI)	Relative Weight (%)
ALL STUDIES					
[23]	160	32–87	-	0.46 (0.22–0.97)	11.81
[22]	203	32–77	66	0.47 (0.29–0.77)	26.55
[25]	190	26–86	61	0.60 (0.40–0.89	41.81
[24]	117	46–80	63	0.37 (0.21–0.66)	19.82
Pooled overall estimate	0.50 (0.38–0.64)				100
Heterogeneity measures	I-squared = 0.0% (*p* = 0.575); Tau-squared = 0.00				
Subgroup Analyses of the Association between VM and OS in HNSCC and ESCC Studies					
**HNSCC STUDIES**					
[22]	203	32–77	66	0.47 (0.29–0.77)	38.84
[25]	190	26–86	61	0.60 (0.40–0.89	61.16
Pooled overall estimate	0.55 (0.40–0.74)				100
Heterogeneity measures	I-squared = 0.0% (*p* = 0.449); Tau-squared = 0.00				
**ESCC STUDIES**					
[23]	160	32–87	-	0.46 (0.22–0.97)	37.33
[24]	117	46–80	63	0.37 (0.21–0.66)	62.67
Pooled overall estimate	0.40 (0.26–0.63)				100
Heterogeneity measures	I-squared = 0.0% (*p* = 0.649); Tau-squared = 0.00				

ESCC, esophageal squamous cell carcinoma; HNSCC, head and neck squamous cell carcinoma; OS, overall survival; VM, vasculogenic mimicry.

## Supplementary Materials

The following are available online at https://www.mdpi.com/2073-4409/9/2/507/s1, Table S1: Evaluation criteria used to assess the quality of the included studies, Table S2: Analysis of the risk of bias of the included studies.
