# Supplementary material for: Vasculogenic Mimicry: A Promising Prognosticator in Head and Neck Squamous Cell Carcinoma and Esophageal Cancer? A Systematic Review and Meta-Analysis
Source: Cells. 2020 Feb 24;9(2):507. doi: 10.3390/cells9020507 (PMC7072765; doi:10.3390/cells9020507)
Supplement: Supplementary file 1 [file cells-09-00507-s001.pdf]

Table S1. Evaluation criteria used to assess the quality of studies.

Checklist Items	Criteria †
**1. Patient samples**	Cohort (retrospective or prospective) study with a well-defined study population with information such as the number of the studied patients, source of sample, study period, follow-up time. Authors explained the medical treatment(s) applied to the patients and clarified if all of the patients received the same treatment or not.
**2. Clinical data of the cohort**	The basic clinical data including gender, age, clinical stage of cancer and histopathological grade, was provided.
**3. Immunohistochemistry**	Well-described staining protocol or referred to original paper with information such as primary antibody name, dilution, company. The cut-off value of the area stained after which it is to be considered positive, was well described.
**4. Prognostics**	The endpoints of the survival analyses were defined (e.g. overall survival, disease-free survival).
**5. Statistics**	Estimated effects (HR, CI) were describing the relationship between the evaluated checkpoint and the outcome was provided. Adequate statistical analysis (e.g. Cox regression modelling) was performed to adjust the estimation of the effect of the biomarker for known prognostic factors.
**6. Classical prognostic factors**	The prognostic value of the classical prognostic factors was reported. The relationship between the evaluated immune checkpoint(s) and classical prognostic factors were reported.

HR, hazard ratio; CI, confidence intervals.

† The criteria was adapted from the REMARK guidelines [20].

Table S2. Analysis of the risk of bias of the included studies †

	Questions ‡											
**Study**	**Q1**	**Q2**	**Q3**	**Q4**	**Q5**	**Q6**	**Q7**	**Q8**	**Q9**	**Q10**	**%**	**Risk of Bias §**
**Liu et al., 2008**	Y	Y	Y	N	U	U	Y	NA	NA	N	50	M
**Wang et al., 2010**	Y	Y	Y	Y	U	Y	Y	NA	NA	Y	87.5	L
**Lin et al., 2012**	Y	Y	Y	Y	U	Y	Y	NA	NA	Y	87.5	L
**Chai et al., 2013**	Y	Y	Y	Y	U	Y	Y	NA	NA	Y	87.5	L
**Zhang et al., 2017**	Y	Y	Y	Y	U	Y	Y	NA	NA	Y	87.5	L
**Wu et al., 2017**	Y	Y	Y	Y	Y	Y	Y	NA	NA	Y	100	L
**Xu et al., 2018**	N	N	N	N	U	Y	Y	NA	NA	N	25	H

† The analysis was performed with the MAStARI (Meta-Analysis of Statistics Assessment and Review Instrument) critical appraisal tool (26).
‡ The questions utilized in this tool were as follows:
Q1. Were there clear criteria for inclusion in the case series?
Q2. Was the condition measured in a standard, reliable way for all participants included in the case series?
Q3. Were valid methods used for identification of the condition for all participants included in the case series?
Q4. Did the case series have consecutive inclusion of participants?
Q5. Did the case series have complete inclusion of participants?
Q6. Was there clear reporting of the demographics of the participants in the study?
Q7. Was there clear reporting of clinical information of the participants?
Q8. Were the outcomes or follow-up results of cases clearly reported?
Q9. Was there clear reporting of the presenting site(s)/clinic(s) demographic information?
Q10. Was statistical analysis appropriate?
§ Risk of bias: L, low 70–100%; M, moderate 50–69%; H, high 49%. The percentage indicates the "yes" score. Y = Yes, N = No, U = Unclear, NA = Not applicable (which was not considered on the percentage calculation). Q8 and 9 were not relevant to the included reports, since an intervention follow-up or an epidemiological distribution of the disease were beyond the aims of these studies.
